# Protocol for the development of a CONSORT-equity guideline to improve reporting of health equity in randomized trials

**DOI:** 10.1186/s13012-015-0332-z

**Published:** 2015-10-21

**Authors:** Vivian Welch, J. Jull, J. Petkovic, R. Armstrong, Y. Boyer, LG Cuervo, SJL Edwards, A. Lydiatt, D. Gough, J. Grimshaw, E. Kristjansson, L. Mbuagbaw, J. McGowan, D. Moher, T. Pantoja, M. Petticrew, K. Pottie, T. Rader, B. Shea, M. Taljaard, E. Waters, C. Weijer, GA Wells, H. White, M. Whitehead, P. Tugwell

**Affiliations:** Bruyère Research Institute, Bruyère Continuing Care and University of Ottawa, 85 Primrose, Ottawa, Ontario Canada; Centre for Health Equity, Melbourne School of Population and Global Health, University of Melbourne, 5/207 Bouverie St Carlton 3010, Victoria, Australia; Canada Research Chair in Aboriginal Health and Wellness, Brandon University, Manitoba, Canada; Research Promotion and Development Office of Knowledge Management, Bioethics and Research Pan American Health Organization, World Health Organization, Washington, DC USA; Research Ethics and Governance, University College London, London, England; Department of Social Science, University College London, London, UK; Ottawa Hospital Research Institute, Medicine University of Ottawa, Ottawa, Canada; School of Psychology, Institute of Population Health, University of Ottawa, Ottawa, Ontario Canada; Cochrane Musculoskeletal Group, London, Ontario Canada; Department of Clinical Epidemiology and Biostatistics, McMaster University, Hamilton, ON Canada; Biostatistics Unit, Father Sean O’Sullivan Research Centre, St Joseph’s Healthcare, Hamilton, ON Canada; Centre for the Development of Best Practices in Health (CDBPH), Yaoundé Central Hospital, Avenue Henri Dunant, Messa, Yaoundé, Cameroon; Department of Medicine, University of Ottawa, Ontario, Canada; Ottawa Hospital Research Institute; School of Epidemiology, Public Health and Preventive Medicine, Faculty of Medicine, University of Ottawa, Ottawa, Canada; Department of Family Medicine, Pontificia Universidad Católica de Chile, Centro Médico San Joaquín Vicuña Mackenna 4686, Macul, Santiago Chile; Department of Social and Environmental Health Research, Public Health Evaluation, Faculty of Public Health and Policy, London School of Hygiene and Tropical Medicine, London, England; Departments of Family Medicine and Epidemiology and Community Medicine Primary Care Research Group and Equity Methods Group, Bruyere Research Institute; School of Epidemiology, Public Health and Preventive Medicine, University of Ottawa, Ottawa, Canada; Canadian Agency for Drugs and Technology in Health, 865 Carling Ave Ottawa, Ontario, Canada; Clinical Epidemiology Program, Ottawa Hospital Research Institute; School of Epidemiology, Public Health and Preventive Medicine, University of Ottawa, Ontario, Canada; Public Health Insight, Melbourne School of Population and Global Health, University of Melbourne, 5/207 Bouverie St Carlton 3010, Victoria, Australia; Rotman Institute of Philosophy, Western University, 1151 Richmond Street, London, Ontario, Canada; Department of Epidemiology and Community Medicine, University of Ottawa, Ottawa, Ontario Canada; Alfred Deakin University, Geelong, Victoria Australia; Department of Public Health and Policy, University of Liverpool, Liverpool, UK

**Keywords:** Randomized controlled trials, Cluster randomized control trials, Health equity, Reporting guidelines, Methods, Health systems, Policy

## Abstract

**Background:**

Health equity concerns the absence of avoidable and unfair differences in health. Randomized controlled trials (RCTs) can provide evidence about the impact of an intervention on health equity for specific disadvantaged populations or in general populations; this is important for equity-focused decision-making. Previous work has identified a lack of adequate reporting guidelines for assessing health equity in RCTs. The objective of this study is to develop guidelines to improve the reporting of health equity considerations in RCTs, as an extension of the Consolidated Standards of Reporting Trials (CONSORT).

**Methods/design:**

A six-phase study using integrated knowledge translation governed by a study executive and advisory board will assemble empirical evidence to inform the CONSORT-equity extension. To create the guideline, the following steps are proposed: (1) develop a conceptual framework for identifying “equity-relevant trials,” (2) assess empirical evidence regarding reporting of equity-relevant trials, (3) consult with global methods and content experts on how to improve reporting of health equity in RCTs, (4) collect broad feedback and prioritize items needed to improve reporting of health equity in RCTs, (5) establish consensus on the CONSORT-equity extension: the guideline for equity-relevant trials, and (6) broadly disseminate and implement the CONSORT-equity extension.

**Discussion:**

This work will be relevant to a broad range of RCTs addressing questions of effectiveness for strategies to improve practice and policy in the areas of social determinants of health, clinical care, health systems, public health, and international development, where health and/or access to health care is a primary outcome. The outcomes include a reporting guideline (CONSORT-equity extension) for equity-relevant RCTs and a knowledge translation strategy to broadly encourage its uptake and use by journal editors, authors, and funding agencies.

## Background

Health equity is defined as the absence of avoidable and unfair differences in health within and between populations [1] and is at the core of global health research priorities [2, 3]. The concept of health equity includes both access to health care as well as the broader concept of opportunities to achieve good health [4–6]. While there is a lack of consensus on the use of the terms health equity, health inequality, and health disparities, we have chosen the term “health equity” [7]. We consider differences in health outcomes across socially stratifying factors such as age, sex/gender, culture, and socioeconomic status to be health inequities when they are considered avoidable and unfair (Table 1). Absent and/or poor quality evidence about health equity is identified by policy makers as a key limitation of research [8, 9]. For example, evidence about poor or black or Hispanic populations is missing in reviews used for drug formulary development in the USA [10]. To address key public health objectives, decision-makers require the best evidence to guide appropriate consideration of the likely effects on health equity in their populations [3, 11, 12].

**Table 1:** Defining health inequity and disadvantage

Health inequalities have been defined as “the virtually universal phenomenon of variation in health indicators … associated with socio-economic status” [50]; inequities may also be seen across other characteristics such as place of residence, ethnicity, gender, etc. Health inequities “are unnecessary, avoidable, unfair, and remediable inequalities” [1, 51, 52]
The characteristics of populations and individuals across which health inequities may exist are multifactorial and may interact with each other. They may also depend on setting and context such as the political climate or health system [17]. Different classification systems have been developed to summarize these characteristics of individuals and populations across which potentially inequitable health differences may exist. Although different factors commonly co-exist, we are using the PROGRESS-Plus organizing framework used by the Cochrane and Campbell Equity Methods Group. The acronym PROGRESS represents: place of residence, race/ethnicity/culture/language, occupation, gender/sex, religion, education, socioeconomic status, and social capital [53, 54]. Additional “Plus” characteristics include (1) individual characteristics (e.g., age, disability), (2) features of relationships (e.g., parents who smoke), and (3) time-dependent transitions (e.g., post-discharge from hospital or prison) [54–56]. Differences in health across these groups do not necessarily denote inequities. For example, increasing cancer incidence with age is not necessarily unfair nor avoidable. However, preferential treatment for younger, fitter people with cancer may be discriminatory [57].
We use the term “disadvantaged” to describe populations who are denied opportunities that others have to benefit from social and environmental conditions that lead to better health. A limitation of the term disadvantaged is that it may be seen as labeling or stigmatizing and it is a term that may not be used by populations or communities to describe their contexts or situations. Many alternative terms are also limited (e.g., underserved or marginalized) because they exclude other population groups. Commitment to health equity is about improving health outcomes for people who have been disadvantaged by social, political, and legal structures, and processes in achieving good health.

Randomized controlled trials (RCTs) are a powerful research design for ascertaining the impact of an intervention and for informing health decisions [13]. There is growing support for well-conducted RCTs to improve the evidence base for the growing field of personalized medicine [14] as well as population and public health [15] and international development [16]. We define RCTs that meet one (or more) of the following criteria as “equity-relevant trials”: (1) assessing effects in a disadvantaged population in relation to a less disadvantaged population (see Table 1), (2) assessing differences in effects between populations considered disadvantaged compared to a less disadvantaged group (see Table 1), or (3) assessing gradient of effects across levels of disadvantage.

There are a number of challenges to overcome if RCTs are to contribute to a robust evidence base for policy making that promotes health equity across populations [17, 18]. There is well-documented under-representation of populations in RCTs who may be disadvantaged due to their ethnicity within a population, or their age, or gender. Even when these groups are included in RCTs, the authors of the trials often fail to report basic sociodemographic details [19] and rarely are subgroup analyses conducted across PROGRESS-Plus characteristics [20, 21]. All too often, this makes it impossible to apply results to populations with particular characteristics or features. Cluster RCTs pose additional challenges when assessing effects on health equity because clusters may include individuals experiencing disadvantage and/or clusters may have different overall experience of disadvantage. Systematic reviews are completely dependent upon the constituent studies, so poor reporting of equity in RCTs is a major constraint on equity assessment in systematic reviews, as has been described frequently but specifically in PRISMA (Preferred Reporting Items for Systematic Reviews and Meta-Analyses)-equity reporting guidelines [22].

### Developing a CONSORT reporting guideline for equity informing trials

The Consolidated Standards for Reporting Trials (CONSORT) statement is an evidence-based guideline consisting of 25 items, intended to encourage completeness and transparency of reporting RCTs. CONSORT has been endorsed by over 50 % of the medical journals indexed in PubMed and has been shown by a systematic review to improve reporting [23]. CONSORT extension statements have been developed for specific issues, such as reporting of cluster RCTs, harms, pragmatic trials, non-pharmacologic therapy, and social and psychological interventions [24]. None of the existing or planned CONSORT extensions addresses reporting characteristics needed to assess the effects of an intervention on health equity (Appendix A).

### Objectives

**Fig 1:**
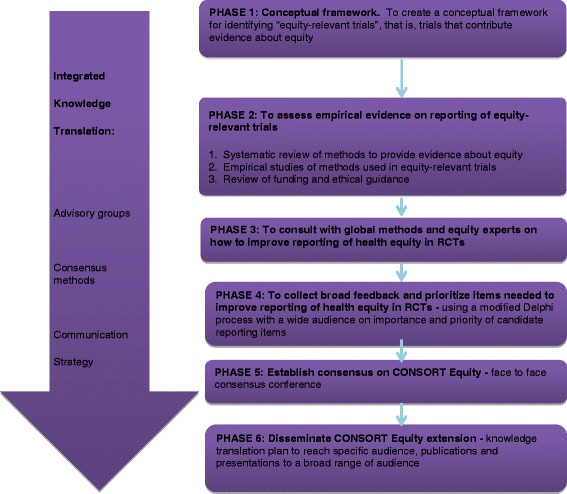
CONSORT-equity: study phases

The main objective of this study is to develop guidelines to improve the reporting of health equity considerations in RCTs, as an extension of CONSORT. This program of research aims to meet the following specific objectives, each of which aligns with a study phase (Fig. 1):

1. To develop a conceptual framework for identifying equity-relevant trials.
2. To assess empirical evidence regarding reporting of equity-relevant trials.
3. To consult with global methods and content experts on how to improve reporting of health equity in RCTs.
4. To collect broad feedback and prioritize items needed to improve reporting of health equity in RCTs.
5. To establish consensus on the CONSORT-equity extension: a reporting guideline for equity-relevant trials.
6. To broadly disseminate and implement the CONSORT-equity extension.

## Methods/design

The study consists of six phases, adapted from the guidance for developing reporting guidelines by Moher et al. [25]. These are identifying the need for the guideline, reviewing the literature, identifying participants, conducting a Delphi survey to gather opinions and set priorities, and holding a face-to-face consensus meeting. There will be two additions to these methods: (1) a review of guidance from research ethics boards and funding agencies about equity, inclusion, and diversity, and (2) consultation using key informant interviews across a broad range of disciplines to gather views on how to improve reporting of effects in subpopulations in RCTs (Fig. 1).

Ensuring the uptake of the CONSORT-equity extension is critical in order to influence reporting of future trials. Therefore, we are using an integrated knowledge translation approach that engages knowledge users as partners throughout the process and as a way to foster thinking about, inclusion, and respect for a multiplicity of perspectives [26]. The co-authors represent a range of disciplines including clinical epidemiology, social science, public health, and international development and are consulted regularly through quarterly meetings. We have developed an international advisory board from intended users across a range of perspectives: journal editors, trialists, bioethicists, patients and members of the public, clinicians, systematic review authors, policy makers, members of disadvantaged populations, and funders (Appendix B). Completion of all study objectives is anticipated by December 2017.

### Phase 1: Development of a conceptual framework

**Fig. 2:**
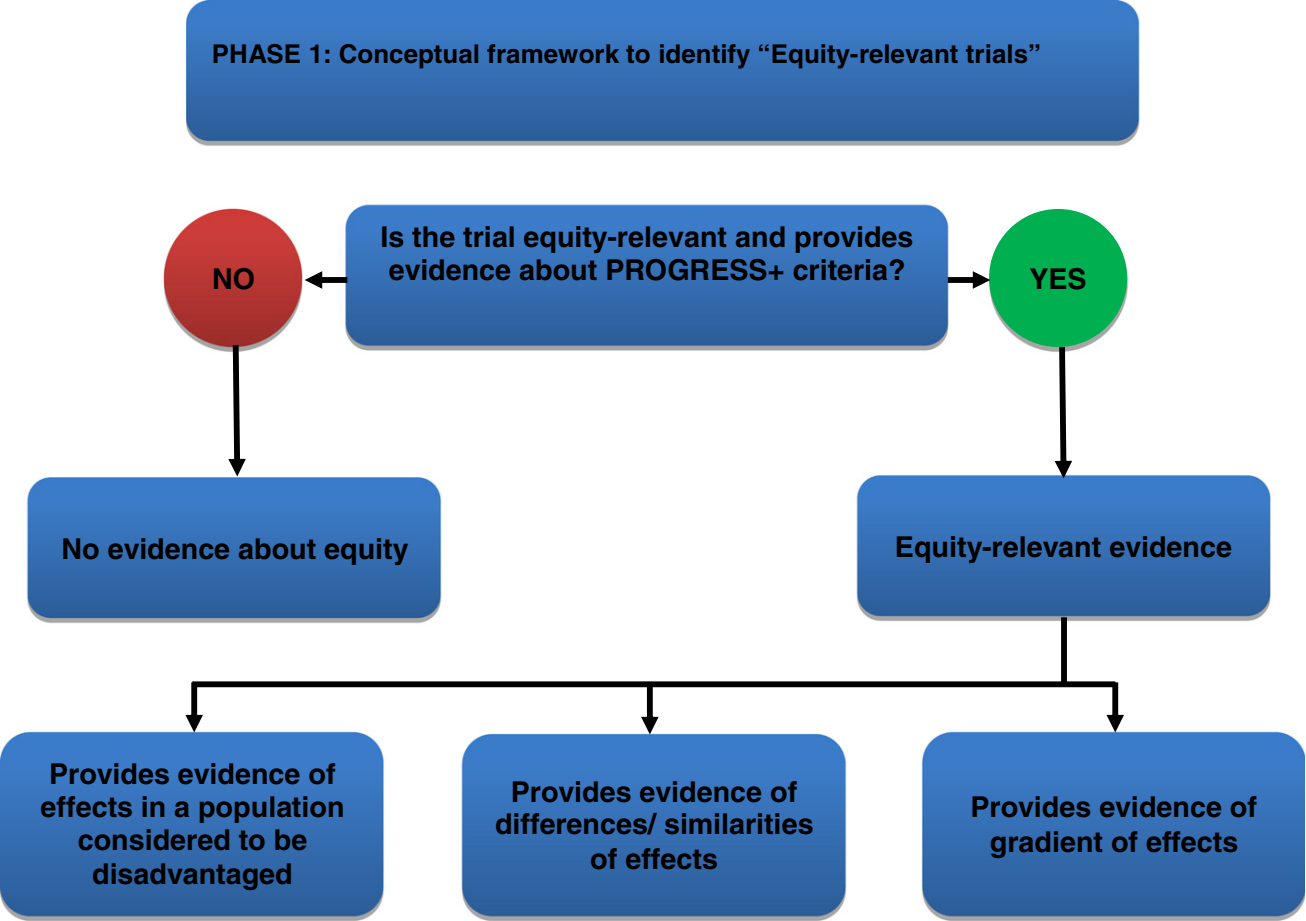
Conceptual framework for identification of equity-relevant trials

We will use an iterative process to develop a conceptual framework for identifying equity-relevant RCTs. To date, we have consulted within the research team and advisory board and reviewed conceptual papers [27–31] to develop a draft conceptual framework (Fig. 2). We tested this framework on sample trials with subgroup analyses across one or more PROGRESS-Plus characteristic and trials conducted in populations who may be disadvantaged in some settings including children, older adults, and individuals with lower income identified by searching PubMed.

We acknowledge that although most health conditions exhibit a gradient of worse health for those experiencing more disadvantage and conventionally described or analyzed using socioeconomic variables such as ethnicity and educational attainment [32], many RCTs have not been powered or designed to provide evidence about health equity; often, feasibility and cost are used as defining reasons. Studies are to be considered for inclusion if they provide estimates of these effects, regardless of the sample or method used to do so. In addition, the appropriateness of sample and method will be assessed as part of the study. We will consider RCTs to be “equity-relevant” if they provide evidence about the following:

1. Effects in a population considered to be disadvantaged,
2. Difference or equivalence in effects across socially stratifying factors, or
3. Gradient of effects across socially stratifying factors.

The first criterion provides direct evidence about health equity by comparing effects across strata of disadvantage. The latter two criteria provide indirect evidence about health equity because there is no comparison to a more advantaged population (Table 2). This framework aligns closely with the framework by Hilary Graham that categorizes three approaches to tackling health inequalities and their implications for evaluation [27]. Furthermore, differences in effects may be assessed by different methods ranging from subgroup analyses to assessing external validity to populations not included in the trial (e.g., many disadvantaged groups are excluded from trials because of restrictive eligibility criteria such as presence of co-morbidities or age).

**Table 2:** Examples of equity-relevant trials and those with no evidence about health equity

Type of trial	Example RCT
1. Provides evidence of effects in a population considered to be disadvantaged	A study of a classroom drama intervention for mental health of immigrant and refugee youth in special classes [58].
	A randomized controlled trial of community-led interventions to prevent domestic violence in Aboriginal communities [59].
2. Provides evidence of difference or equivalence in effects across socially stratifying factors	A study of sex differences in platelet reactivity and cardiovascular and psychological response to mental stress in patients with stable ischemic heart disease [60].
	A study of the impact of an informed choice invitation on uptake of screening for diabetes in primary care assessed whether there were differences in effectiveness according to socioeconomic status [61].
3. Provides evidence of a gradient of effects across socially stratifying factors	A study of individual- and area-level unemployment influence smoking cessation among African Americans [62].
4. Provides no evidence about equity	Efficacy of the transillumination method for appropriate tracheal tube placement in small children: a randomized controlled trial [63].
	A comparison of cast materials for the treatment of congenital idiopathic clubfoot using the Ponseti method [64].

We will test the clarity, acceptability, and feasibility of using this conceptual framework by discussing sample trials with three types of stakeholders:

1. Advisory board members (Appendix B)
2. Investigators of effectiveness trials committed to informing equity
3. Community representatives from disadvantaged populations

This may result in additional changes to the conceptual framework.

### Phase 2: Assessing the evidence

We will address three research questions about the strengths and limitations of methods used to provide evidence about equity in RCTs as well as existing guidance regarding the use of these methods:

1. What are the strengths and limitations of methods used in equity-relevant trials?
2. What is the existing guidance on conducting equity-relevant trials?
3. What is the quality of reporting of equity-relevant trials?

#### Phase 2, question 1: What are the strengths and limitations of methods used in equity-relevant trials?

We will conduct a Cochrane methodology systematic review to assess the strengths and limitations of methods used to address equity in equity-relevant trials. A methodology review is different from an intervention review since it aims to assess the strengths and limitations of methods used for research rather than the impact of interventions. For example, Welch and colleagues used a similar approach to assess the strengths and limitations of methods to assess equity in systematic reviews [33]. We will develop an a priori protocol, following Cochrane Handbook methods [34] and Cochrane Methodology Review Group guidance [35]. The eligible studies will be empirical studies of methods used in equity-relevant trials. We have identified examples of eligible studies in Table 3. We will design a search for evidence with a librarian scientist (JM) encompassing electronic databases (e.g., MEDLINE, The Cochrane Library, Cochrane Methodology register, Sociological abstracts) and gray literature, using a combination of text words and subject headings, and assess its ability to identify a reference set of known articles (Appendix C). We will also use Web of Science to search for studies that cite eligible studies [36]. The protocol will be submitted for publication in The Cochrane Library. The results of this review will contribute candidate items for reporting equity-relevant trials.

**Table 3:** Examples of eligible studies for Cochrane systematic review of methods to assess equity

1. Recruitment methods to engage disadvantaged populations in trials
• Study of the effect of incentive payments in 5 RCTs on recruitment of ethnic minorities [65]
2. Reporting population characteristics
• Study of 100 RCTs in four leading medical journals assessed for reporting of sociodemographic characteristics in “Table 1” [19]
3. Subgroup analyses
• Study of 169 cardiovascular RCTs for quality of reporting and conducting sex/gender subgroup analyses [66]
4. Applicability of evidence
• Study of reasons for exclusion of participants from RCTs and effects on applicability decisions [39]

#### Phase 2, question 2: What is existing guidance on conducting equity-relevant trials?

We will conduct a review of existing guidance for equity-relevant trials such as inclusion of vulnerable populations and ethical concerns. This will include guidance developed by any type of organization, such as academic groups (e.g., CONSORT), funding agencies (e.g., NIH guidance on inclusion of women and minorities), governmental and non-governmental organizations (e.g., Institute of Medicine guidance on sex-specific reporting in research [37]), and the National Guidelines Clearinghouse (www.guideline.gov). We will design a search strategy according to the Peer-Reviewed Electronic Search Strategies (PRESS) guideline [36] that includes both electronic databases and targeted search of websites of relevant organizations with an experienced librarian scientist (JM). Websites of organizations that provide ethics guidance will be searched (e.g., Institute of Medicine, Council for International Organizations of Medical Sciences and World Association of Medical Editors), as well as conducting open web searches.

Data will be extracted using a pretested form on the methods of developing the guidance, values statements (if provided), items related to study design, inclusion and protection of different disadvantaged populations, and any evidence on validation, uptake, or use of the guidelines. We will assess the extent to which populations defined as disadvantaged across PROGRESS-Plus criteria are considered in the guidance. The items identified by this review will be considered for inclusion in CONSORT-equity.

#### Phase 2, question 3: What is the quality of reporting equity-relevant trials?

We will conduct an empirical review of a random sample of published equity-relevant trials to assess methodological quality and reporting of equity considerations. We will develop a search strategy for equity-relevant trials, in collaboration with a librarian scientist (JM), using both text words and MeSH headings, and test this search with a reference set of equity-relevant trials. We will search MEDLINE, Sociological abstracts, and Econlit to encompass medical, public health, and international development interventions. We will restrict the search to the last 3 years since reporting has improved over time, and we expect a sufficient number of RCTs in this period to meet our criteria. Two independent reviewers will screen titles and abstracts for eligibility using the conceptual framework to identify equity-relevant trials. We will select a random sample of 100 individually randomized trials and 100 cluster RCTs. We decided to balance the sample according to cluster versus individual randomization to ensure an adequate number of each design, because cluster RCTs have unique equity considerations and because they may require different analysis strategies [38]. We will extract data regarding the study design, populations, and analysis across PROGRESS-Plus characteristics using a pretested data extraction form, with two independent reviewers. We will assess reporting of methods using accepted criteria. For example, we will assess the extent to which subgroup analyses meet criteria for credibility [34]. We expect that the most commonly used methods will be subgroup analyses, and if these are used by 25 % of trials, each sample of 100 trials will provide a margin of error of ±8.5 % around this proportion. We will describe the range of methods used to provide evidence about equity (e.g., subgroup analysis, comparison of trial populations with target population to judge external validity [39]), any comparison between methods (if done), and quality of reporting or conduct for specific methods (e.g., quality of subgroup analyses). The results will be used to identify candidate items for the CONSORT-equity guideline.

### Phase 3: To consult with global experts on candidate items to improve reporting of equity-relevant trials

Health equity issues span a breadth of disciplinary fields that may have different approaches to reporting trials. Because we aim for broad relevance of this reporting guideline, we want to seek opinions regarding how to improve reporting of equity-relevant trials from different disciplinary perspectives. We will conduct key informant, semi-structured interviews since this is an efficient way to engage diverse stakeholders, and then collect and synthesize their views using thematic analysis. We will use results of the empirical studies (phase 2) to design a semi-structured interview guide. The interview guide will be designed to invite feedback on candidate items identified in the prior studies as well as seeking new items.

We will identify participants through our co-authors and advisory board as well as lead authors of empirical studies and other guidance (e.g., ethics, funding agencies). We will select participants to maximize variation of disciplines and stakeholder organizations (e.g., academic, non-governmental, research ethics boards, governmental). We will conduct interviews by phone or face to face and take notes during the interviews as well as tape record interviews. We will expand our sample by snowball sampling: an approach to recruit participants that builds on networks by asking each participant to suggest additional contacts [40]. Sample size will be determined by theoretical saturation, defined as when subsequent interviews contribute no new data, and is estimated to occur at 10–13 interviews [41]. Thematic analysis of transcribed interviews will be conducted by two coders, using NVivo qualitative software to facilitate analysis [42]. This portion of the study has been submitted to the Bruyère Research Institute Ethics Board for approval.

### Phase 4: To prioritize candidate items for reporting guideline

Prior to seeking consensus (phase 5), it is important to seek external feedback on the importance of different candidate items identified in the preceding empirical studies [25]. In this phase, we aim to prioritize items and invite feedback on proposed items. We will use the Delphi process for this prioritization because it is a structured, iterative approach to obtaining information by posing a series of questions to a select group of experts in the area for which the information is sought [43]. To identify participants, we will use electronic mailing lists and social media to reach members of the intended users of the reporting guideline, such as trialists, methodologists, clinicians, and decision-makers. Patient engagement and citizen participation is critical from an equity perspective since patients and citizens are the intended beneficiaries of the results of trials. Patient and citizen views will be sought by engaging with networks of patients and citizens such as the Cochrane Consumer Network (led by AL, who is the consumer representative on the Cochrane Steering Group).

We will present a preliminary list of candidate items, with examples, and ask participants to rank their importance using an online survey tool. We will invite open-ended comments and suggestions for new items. We plan to conduct up to three rounds of an online Delphi survey, as for previous CONSORT guidelines [44–46]. If consensus is reached earlier, we will conduct fewer rounds of the survey. This study has been submitted to the Bruyère Research Institute Ethics Board for approval.

### Phase 5: Consensus development

As recommended by Moher et al. [25], we will hold a 2-day face-to-face consensus meeting to engage and build consensus with users of the reporting guideline. We will design this meeting to facilitate engagement and equitable opportunities for contributions from all conference participants, by assigning roles as chairs, facilitators, discussants, and rapporteurs to each participant, as used previously by Welch et al. [22]. Participants will include members of the research team and advisory board and may include other external stakeholders.

During the meeting, we will present the results for each candidate item from the empirical studies and the Delphi process with examples and use a structured discussion to reach consensus on included items. The discussions will be audio-recorded and transcribed verbatim. As with other consensus meetings, word crafting will be left until post-meeting. We will use meeting transcripts and notes to finalize the CONSORT-equity reporting guideline.

Next, we will conduct an iterative process of usability testing of the guideline by intended users to assess clarity and acceptability of items. Written and oral feedback from these users will be incorporated. Consensus group members will participate in an iterative process of crafting the final guideline. We will develop an elaboration and explanation document with exemplars of good reporting and details of empiric evidence to support each item. This study has been submitted to the Bruyère Research Institute Ethics Board for approval.

### Phase 6: Dissemination and implementation

We will develop a knowledge translation (KT) plan at the consensus conference, led by JMG, a known expert in the area of implementation science and KT. We will design strategies to promote implementation of the reporting guideline by thought leaders who publish, use, fund, or conduct RCTs across different disciplines. Possible strategies may include training workshops at events, such as at the Cochrane Colloquia and the Peer Review Congress, and webinars that will be made available on open-access websites such as the Campbell and Cochrane Equity Methods group website [47]. To reach journal editors and funders, we expect to use targeted approaches such as direct letters with specific messages (e.g., how to include this reporting guideline in instructions for authors and applicants). Throughout the study, we will use information technology to raise awareness, maintain a list of publications, and invite comments through an open-access website [48], writing blogs, and the use of social media, including twitter [49].

## Discussion

Following the WHO Commission on the Social Determinants of Health (CSDH), there is increasing interest in health equity [17]. However, variable or inadequate reporting of data from trials which can inform health equity decisions could contribute to waste in research and may not serve the needs of people who are experiencing health inequities. This program of research is the first known attempt to improve the reporting of health equity in RCTs and has been designed to complement current and in-development CONSORT statement extensions. Improved reporting of equity considerations is relevant to health research funders, decision-makers, and practitioners who use evidence from RCTs to inform decisions, researchers conducting equity-oriented systematic reviews, and civil society who benefit from these decisions. We hope this reporting guideline will contribute to an improved evidence base for equity-oriented decisions and promote a broader agenda in relation to health equity.

## Appendix B: CONSORT-equity advisory board members

### CONSORT-equity advisory board

1. Yvonne Boyer
   Canada Research Chair in Aboriginal Health and Wellness
   Brandon University
2. Luis Gabriel Cuervo
   Senior Advisor for Research Promotion and Development, PAHO
3. Sarah Edwards
   University College London
4. Caroline Kisia
   Executive Director, Action Africa Health-International
5. Anne Lydiatt
   Patient partner, Canadian Institutes of Health Research Strategy for Patient Oriented Research
6. Lawrence Mbuagbaw
   Assistant Professor, Epidemiology and Biostatistics, McMaster University
   Centre for Development of Best Practices in Health, Yaoundé Central Hospital, Cameroon
7. Janet Smylie
   CIHR Applied Public Health Chair and Director, Well Living House Action Research Centre for Indigenous Infant, Child and Family Health and Wellbeing, St. Michael’s Hospital and DLSPH, University of Toronto
8. Jimmy Volmink
   Dean, Centre for Evidence-Based Health Care, Faculty of Medicine and Health Sciences, Stellenbosch University and Cochrane South Africa, South African Medical Research Council, Cape Town, South Africa
9. Margaret Whitehead
   WH Duncan Professor of Public Health, Division of Public Health, School of Population, Community and Behavioural Sciences, The University of Liverpool

## Appendix C: Search strategy to identify equity-relevant trials

1. exp Gender Identity/
2. (gender-based or gender-related or gender factors).tw.
3. ((sex or gender) adj3 (analysis or factor$ or inequit$ or disparit$ or inequalit$ or difference$ or interact$)).mp. [mp=title, abstract, original title, name of substance word, subject heading word, keyword heading word, protocol supplementary concept word, rare disease supplementary concept word, unique identifier]
4. exp sex factors/
5. exp geriatrics/
6. ((ethnic$ or race or racial or religio$ or cultur$ or minorit$ or refugee or indigenous or aboriginal or African american) adj3 (analysis or disparit$ or inequalit$ or inequit$ or difference$ or predict$ or interact$)).tw.
7. exp homosexuality/
8. exp disabled persons/
9. ((poverty or low-income or “lower income” or socioeconomic$ or socio-economic or social) adj3 (analysis or disadvantage$ or factor$ or inequalit$ or depriv$ or inequit$ or disparit$ or difference$ or predict$ or interact$)).tw.
10. exp Educational Status/
11. exp Socioeconomic Factors/
12. ((discriminat$ or social exclu$ or social inclu$) adj3 (religion or culture or race or racial or aboriginal or indigenous or ethnic$)).tw.
13. ((urban or rural or inner-city or remote or slum) adj3 (analysis or inequit$ or disparit$ or inequalit$ or difference$ or predict$ or interact$)).tw.
14. ((resource-poor or (“low income” adj countr$) or (“middle income” adj countr$) or africa or developing countr$ or “south america” or china or asia or “latin america”) adj3 (relevance or analysis or applicab$ or inequit$ or disparit$ or inequalit$ or difference$ or predict$ or interact$)).tw.
15. (inequalit$ or in-equalit or equit$ or inequit$ or in-equit or disparit$ or underserved or marginali$ed).tw.
16. exp indigenous populations/
17. ((native* or Indian or aborigin*) adj3 (American* or Canadian* or Alaska*)).tw.
18. (first adj2 nation*).tw.
19. (aborigin$ or metis or inuit$ or eskimo$ or native or esquimaux or aleut or yuit or inughuit or unanga* or alutiiq or inup#ia* or kalaallit or Inuktitut or Nunavut or nunavik or cree or dene or haida or salish or Mohawk or ojibway or yupik or tribal or arctic).tw.
20. exp american native continental ancestry group/ or oceanic ancestry group/
21. exp rural health/
22. or/1–21
23. randomized controlled trial.pt.
24. (randomized or placebo).mp.
25. (cluster$ adj2 randomi$).tw.
26. or/23–25
27. 22 and 26
